# Infrared Lightwave Memory-Resident Manipulation and Absorption Based on Spatial Electromagnetic Wavefield Excitation and Resonant Accumulation by GdFe-Based Nanocavity-Shaped Metasurfaces

**DOI:** 10.3390/nano14141230

**Published:** 2024-07-20

**Authors:** Cheng Chen, Chuang Zhang, Taige Liu, Zhe Wang, Jiashuo Shi, Xinyu Zhang

**Affiliations:** 1 National Key Laboratory of Science and Technology on Multispectral Information Processing, Huazhong University of Science and Technology, Wuhan 430074, Chinam202373645@hust.edu.cn (C.Z.); wangzhe9836@163.com (Z.W.); 2 School of Artificial Intelligence and Automation, Huazhong University of Science and Technology, Wuhan 430074, China; 3 Faculty of Information Engineering and Automation, Kunming University of Science and Technology, Kunming 650500, China; shijiashuo97@163.com

**Keywords:** nanocavity-shaped metasurface, GdFe-based film system configuration, electromagnetic excitation, resonant accumulation, spatial magnetic plasmons, lightwave memory-resident manipulation and absorption

## Abstract

An arrayed nanocavity-shaped architecture consisting of the key GdFe film and SiO_2_ dielectric layer is constructed, leading to an efficient infrared (IR) absorption metasurface. By carefully designing and optimizing the film system configuration and the surface layout with needed geometry, a desirable IR radiation absorption according to the spatial magnetic plasmon modes is realized experimentally. The simulations and measurements demonstrate that GdFe-based nanocavity-shaped metasurfaces can be used to achieve an average IR absorption of ~81% in a wide wavelength range of 3–14 μm. A type of the patterned GdFe-based nanocavity-shaped metasurface is further proposed for exciting relatively strong spatial electromagnetic wavefields confined by a patterned nanocavity array based on the joint action of the surface oscillated net charges over the charged metallic films and the surface conductive currents including equivalent eddy currents surrounding the layered GdFe and SiO_2_ materials. Intensive IR absorption can be attributed to a spatial electromagnetic wavefield excitation and resonant accumulation or memory residence according to the GdFe-based nanocavity-shaped array formed. Our research provides a potential clue for efficiently responding and manipulating and storing incident IR radiation mainly based on the excitation and resonant accumulation of spatial magnetic plasmons.

## 1. Introduction

With the rapid development of current micro-nano-techniques, many types of metasurfaces are already constructed by arrays integrating basic nano-architectured elements such as nanoholes, nanocylinders, nanostripes [1], or nanodisks [2], as well as patterned nano-composites from common ferroelectric or ferromagnetic materials involving some noble metals. Generally, several functional metasurfaces still need to be configurated by layered semiconductive or dielectric films based on surface or spatial plasmon excitation and oriented transportation and localized re-arrangement or resonant residency, according to featured electromagnetic wavefield modes induced by incident lightbeams on the basis of essential energy [3] and a momentum conservation mechanism [4]. As shown, a resonant generation and ordered distribution of plasmon polaritons can be selectively manipulated by particularly configurating or carefully modifying the micro-nano-layout with a suitable parameter option [5], such as the radius and depth of typical nanoholes or nanocylinders and their arrangement period. So, an arrayed micro-nano-architecture can be particularly configurated by an intermediate film or film system, leading to a type of resonant cavity for holding more electromagnetic wavefields than those only attached on a special surface or interface [6]. According to traditional concepts, the magnetic field component of lightwaves can be efficiently compressed into a relatively large space or a magnetic medium pipeline or cavity, but the electric field component should be inductively constrained by a two-dimensional facet based on the surface “free electron” excitation and transportation and arrangement according to surface plasmon modes [7].

As demonstrated, many ferromagnetic materials can exhibit a relatively powerful magnetic response induced by a weak and guided magnetic field such as an oscillated magnetic component [8] of incident lightwaves [9]. Considering the common situation of the magnetic energy state far lower than the electric field of lightwaves in a wide wavelength range including visible light and infrared (IR) radiation, an accumulating enhancement in the magnetic fields in a sealed or a semi-opened shallow cavity with a suitable geometry can be more easily achieved or further efficiently manipulated corresponding to the electric field component coupled closely with them. It should be noted that resonant magnetic field stockpiling will be more effectively realized by a typical micro-nano-cavity according to modern optoelectronic technologies [10]. So, a recent hotspot is already focused on how to construct novel nano-architectures to generate strong magnetic plasmon resonance so as to greatly enhance the collecting efficiency of incident lightwaves, which also means a remarkable prompting of the capturing ability of incident radiation. As shown, incident lightwaves can be used to stimulate strong magnetic plasmon oscillations similar to traditional waveguide modes [11] in a typical layered magnetic film system [12]. Due to the concurrent presence of the surface plasmons excited and thus a strong magnetic response leading to a tremendous enhancement in magnetic induction intensity in a particular magnetic medium cavity [13], the coupled electric field strength from the same functional structures can also be significantly amplified [14] and thus enable to more efficiently collect incident light energy [15,16].

Considering the situation of IR radiation absorption basically being based on the magnetic nanocavity of the metasurface developed by us, a common GdFe as a typical soft magnetic material has been selected as the main functional material for effectively responding to a rapid variance in incident lightwaves. In general, optical metasurfaces based on the specific construction of the metal–insulator–metal architecture can be used to achieve an effective narrow or broadband radiation absorption. However, a broadband-absorbing metasurface may play an important role in energy harvesting applications, leading to a multilayer stacking approach. So, a kind of metasurface based on the GdFe-based nanocavity presented in this paper is formed by vertically cascading two GdFe-SiO_2_ nanocavities. The GdFe films of 100 nm and 50 nm as well as the SiO_2_ films of 900 nm and 500 nm are already layered and configurated over each Ag film of 10 nm in the nanocavities. As an IR memory-resident absorption metasurface (IMAM), it can be potentially employed to sufficiently capture incident lightwaves in a wide wavelength range far more than in the visible range, for instance, realizing full spectral solar power generation according to the radiation characteristics of the sun, achieving full-time photovoltaic generation through receiving the IR radiation of the earth [17] as another “sun” to illuminate all objects on the earth at night, or even applying it in the IR stealth [18] field [19]. At first, the main characteristics of the IMAM developed by us are simulated for efficiently performing surface plasmon excitation and then short-range transportation and thus localized resonance enhancement. And a type of nanocavity-shaped metasurface is designed and devised for a strong resonant accumulation of the spatial magnetic fields also closely correlated with a strong spatial electric field distribution, which can be viewed as a memory-resident radiation absorption mechanism apparently different from the conventional photothermal absorption. Finally, the key IR absorption characteristics of IMAMs with different facial configuration are evaluated experimentally. Current research indicates that the IR response of IMAMs can be improved remarkably through carefully tuning the morphology of the electromagnetic wavefields existing in a patterned micro-nano-architecture. At present, a strong spectral IR absorption of nearly 100% is already realized in a wide wavelength range of 3–14 μm, only through the layered stacking of both the GdFe film and SiO_2_ layer, leading to an arrayed dual nanocavity-shaped metasurface, which is filled fully or memorably resided by excited electromagnetic plasmons. And the joint action of the surface oscillated net charges over the charged metallic films and the surface conductive currents including equivalent eddy currents surrounding the layered GdFe and SiO_2_ materials is also available to interpret the IR absorption of the patterned GdFe-based nanocavity-shaped metasurface.

## 2. Materials and Methods

**Figure 1:**
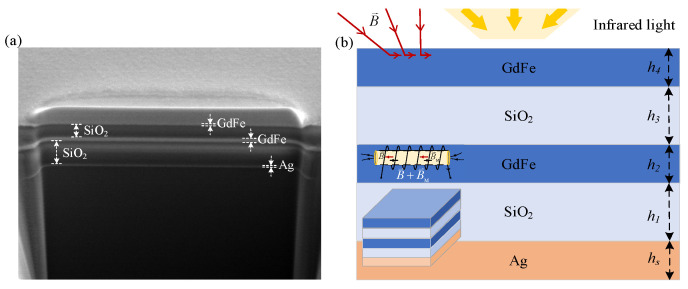
A dual nanocavity-shaped metasurface shaped by vertically cascading two basic GdFe-SiO_2_ nanocavities. (**a**) A SEM photograph of a fabricated cross-sectional metasurface sample. (**b**) Layered configurating GdFe films with *h*_2_ and *h*_4_ thickness and SiO_2_ layers with *h*_1_ and *h*_3_ thickness over an Ag film with *h*_s_ thickness, respectively.

The GdFe-based nanocavity-shaped metasurfaces proposed by us are basically constructed through alternately depositing a GdFe film and SiO_2_ dielectric layer, as shown in Figure 1. The transmission coefficient of the proposed metasurfaces can be expressed as $S_{21}=[\sin(nkd)-\frac{i}{2}(Z+\frac{1}{Z})\cos(nkd)]e^{ikd}$, where the parameter *d* is the thickness of the metasurface, and $n=n_{1}+in_{2}$ denotes the complex refractive index of the metasurfaces, *Z* its impedance, and *k* the wave-vector of the incident lightwave. When the substrate is thick enough, the transmission coefficient will approximate 0. By finely adjusting the key parameters, including the thickness and the material composition, several factors such as the phase and the amplitude and the transmissivity of incident IR light can be precisely manipulated. As known, a periodic overlapping of the medium films stacked between two metallic films will result in a functioned dual nanocavity-shaped film system for sufficiently suppressing or even completely eliminating [20] the reflection loss of incident electromagnetic wavefields [21] through the phase cancellation operation [22], such as typical beam destructive interference.

As shown in Figure 1, a dual nanocavity-shaped metasurface is shaped by vertically cascading two basic GdFe- SiO_2_ nanocavities. The upper nanocavity consists of a top and bottom GdFe film and an intermediate SiO_2_ layer and the lower by a top GdFe film (also the bottom GdFe film of the upper nanocavity) and an intermediate SiO_2_ layer and a Ag film over an n-type Si wafer, which are fabricated by a traditional film system growing flow. A cross-sectional view of the fabricated IMAM sample is exhibited by a SEM photograph shown in Figure 1a. The film system parameters are expressed by a set of the film thickness of {*h*_1_—*h*_4_,*h*_s_}, where the depth of the upper nanocavity is *h*_2_ + *h*_3_ + *h*_4_ and the lower by *h*_1_ + *h*_2_. The yellow IR lightwaves including typical perpendicular and inclined beams are incident upon the surface of the metasurface constructed. Generally, the GdFe material can be intensively magnetized by a guiding magnetic field being similar to a common solenoid with a ferromagnetic core so as to present a total magnetic induction intensity of **B + B**_M_, which is usually far more than the guiding magnetic induction intensity **B** colored in red. Based on the general mechanism, the incident magnetic induction lines slanted upon the surface of a magnetic material will almost completely enter into the magnetic medium along its internal surface, because of a tremendous difference between the magnetic medium and circumstance, as shown in Figure 1b. So, an almost entire magnetic field component of the incident beams can be guided into a magnetic surface or interface, generally presenting an enhancement of more than 3 orders of magnitude. The above effect also means that there is a transient surface electric field response of the magnetic medium because of a close coupling between the transient electric field and the magnetic field components according to traditional Maxwell electromagnetic relation, but a certain extent phase retard is usually exhibited between them such as a maximum π value or an antiphase state.

According to the layout design of the IMAM, a relatively strong magnetic induction intensity will be generated in the top and also the intermediate GdFe films from the guiding magnetic field component of the incident lightwaves already crossing the incident surface and further the lower interfaces so as to effectively re-orient the micromagnetic domains in GdFe films. It can be expected that the surface plasmons will also be excited mainly over the top surface of the first GdFe film, thus resulting in a surface “free electron” oscillation and then transportation and thus redistribution according to the surface “free electron” density wave, simultaneously. The action above will be further transferred onto the next metallic film to continuously arouse the surface induction electric current fields and also the inevitable Joule loss of the IR radiation within a specific wavelength range [23]. So, a detailed analysis and evaluation about the memory-resident or resonant IR absorption characteristics of the IMAM are as follows.

**Figure 2:**
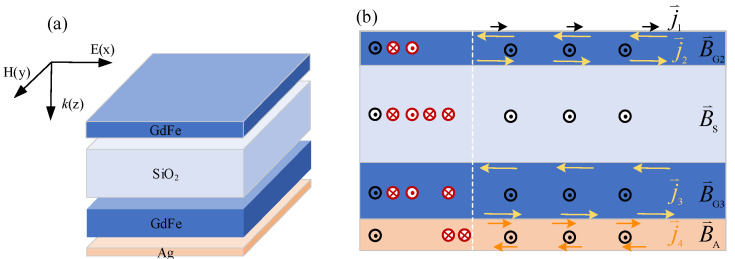
A symbiotic architecture for generating the layered magnetic fields distributed in each functional film labeled by two arrows of entering and exiting the paper, the surface electric current fields including the black surface current **J**_1_ of the transient surface plasmons excited by the incident radiations, two similar yellow eddy currents **J**_2_ and **J**_3_ over the top and bottom endfaces of a single GdFe film, and the similar brown eddy current **J**_4_ over two endfaces of the bottom Ag film, respectively. (**a**) A basic configuration based on GdFe and SiO_2_ and Ag films leading to a nanocavity-shaped architecture for responding to the IR radiations according to the transverse electric field (TE) and the transverse magnetic field (TM) incidence, where the time-varying TM component labeled by the black arrows of exiting the paper will penetrate the film system. (**b**) Overlapping the black penetrating magnetic fields and the layered magnetic field components labeled by two red arrows of entering and exiting the paper, which are excited by the surface equivalent eddy current above to form the net resonant magnetic fields according to the spatial magnetic plasmon modes.

A symbiotic architecture for resonantly forming a set of layered spatial magnetic fields distributed in each functional film is shown in Figure 2. A basic configuration based on GdFe and SiO_2_ and Ag materials leading to a nanocavity-shaped scheme for responding to the IR radiations according to the transverse electric field (TE) and the transverse magnetic field (TM) incidence is shown in Figure 2a. The orientation of both the TE and TM waves are parallel to the surface of the top magnetic film, but the wave-vector **k** is perpendicular to it. As shown in Figure 2b, the time-varying TM component labeled by the black arrows of exiting the paper will penetrate the film system. The other magnetic field components labeled by two types of arrows of entering and exiting the paper are stimulated simultaneously by the surface electric current fields including the black surface current **J**_1_ of the transient surface plasmons excited by incident radiations, two similar yellow eddy currents **J**_2_ (**J**_2U_, **J**_2B_) and **J**_3_ (**J**_3U_, **J**_3B_) over the top and bottom endfaces of a single GdFe film, and a similar brown eddy current **J**_4_ (**J**_4U_, **J**_4B_) over two endfaces of the bottom Ag film, respectively, where (**J**_2U_, **J**_2B_) and (**J**_3U_, **J**_3B_) and (**J**_4U_, **J**_4B_) denote the currents distributed over the upper and lower endfaces of the films above. So, an initial invariant **H** of the incident IR beams will penetrate the film system in the nanocavity and thus guide the formation of the spatial magnetic field in each film. Through overlapping the black penetrating magnetic fields and the layered magnetic field components labeled by two types of red arrows of entering and exiting the paper excited by the surface currents and the similar eddy currents, as a sequence of {(**J**_1_)@**B**/**B**_S_/**B**_G3_/**B**_A_ + (**J**_2U_,**J**_3B_∪**J**_4U_)@**B**_G2_/**B**_S_/**B**_G3_ + (**J**_2B_,**J**_3U_)@**B**_S_ + (**J**_2B_,**J**_4B_)@**B**_S_/**B**_G3_/**B**_A_ + (**J**_4U_,**J**_4B_)@**B**_A_}, a net spatial magnetic field can be constructed in the dual nanocavity according to a spatial magnetic plasmon resonance (MPR) mode. Since the IR loss based on the MPR is much smaller than the LSP formed mainly according to the surface electric field resonance, a very strong magnetic field resonance induced mainly in the dual nanocavity can be generated, which also means a significant strengthening of the spatial electric fields closely associated with the magnetic fields so as to result in a spatial standing wave enhancement or a memory-resident absorption of high-energy state light fields.

Considering the relative magnetic permeability of the magnetic material utilized, a *z* = 0 plane is selected as an incident surface of IR radiation. A basic wave equation of $\frac{\partial E(z)}{\partial z^{2}}+(k_{0}^{2}\varepsilon \mu -k^{2})E=0$ and the relations of $\vec{e_{n}}\times (\vec{D_{2}}-\vec{D_{1}})=\sigma$ and $\vec{e_{n}}\times (\vec{H_{2}}-\vec{H_{1}})=\alpha$ are used to describe the transportation behaviors in a layered film system, where σ and α are the surface net charge density and the surface conductive current density, respectively. According to the situation without any surface conductive current over both the GdFe and Ag films, a continuity of Hx and Hy can be expected. Based on the momentum conservation over the incident surface, a relation of $\frac{k_{1}}{\varepsilon_{1}}+\frac{k_{1}}{\varepsilon_{2}}=0$ can also be induced. In order to obtain the guided wavefield modes confined near the interface, the wave-vectors perpendicular to the interface of the metallic and dielectric materials must present an opposite orientation, for example, Re[*k*_1_] > 0 and Re[*k*_2_] > 0. By substituting the Hy component into the wave equation above, an asymptotic impedance matching can be acquired as $k=k_{0}\sqrt{\frac{\varepsilon_{1}^{2}\varepsilon_{2}\mu_{2}-\varepsilon_{1}\varepsilon_{2}^{2}\mu_{1}}{\varepsilon_{1}^{2}-\varepsilon_{2}^{2}}}=k_{0}\sqrt{\frac{\varepsilon_{1}\varepsilon_{2}}{\varepsilon_{1}+\varepsilon_{2}}\frac{\varepsilon_{1}\mu_{2}-\varepsilon_{2}\mu_{1}}{\varepsilon_{1}-\varepsilon_{2}}}$. When *z* > 0, an important relation of $k_{2}^{2}=k-k_{0}^{2}\varepsilon_{2}\mu_{2}=\frac{k_{0}^{2}\varepsilon_{2}^{2}}{\varepsilon_{1}^{2}-\varepsilon_{2}^{2}}(\varepsilon_{2}\mu_{2}-\varepsilon_{1}\mu_{1})$ can be obtained in the dielectric region, and thus both the electric field and magnetic field components can be expressed, respectively, as

(1) $$E_{x}(z)=iA_{2}\frac{1}{\omega \varepsilon_{0}\varepsilon_{d}}k_{2}e^{ikx}e^{-k_{2}z},$$

(2) $$E_{z}(z)=-A_{2}\frac{1}{\omega \varepsilon_{0}\varepsilon_{d}}e^{ikx}e^{-k_{2}z},$$

(3) $$H_{y}(z)=A_{2}e^{ikx}e^{-k_{2}z},$$

And in the metal region, a relation of $k_{1}^{2}=k-k_{0}^{2}\varepsilon_{1}\mu_{1}=\frac{k_{0}^{2}\varepsilon_{1}^{2}}{\varepsilon_{1}^{2}-\varepsilon_{2}^{2}}(\varepsilon_{2}\mu_{2}-\varepsilon_{1}\mu_{1})$ can be induced, and further, both the electric field and magnetic field components are

(4) $$E_{x}(z)=iA_{1}\frac{1}{\omega \varepsilon_{0}\varepsilon_{m}}k_{2}e^{ikx}e^{-k_{1}z},$$

(5) $$E_{z}(z)=-A_{1}\frac{1}{\omega \varepsilon_{0}\varepsilon_{m}}e^{ikx}e^{-k_{1}z},$$

(6) $$H_{y}(z)=A_{1}e^{ikx}e^{-k_{1}z},$$

To non-magnetic media, the surface plasmon wave-vector **k** should be $k=k_{0}\sqrt{\frac{\varepsilon_{1}\varepsilon_{2}}{\varepsilon_{1}+\varepsilon_{2}}}$. Due to the radiation absorption that happened between the Si substrate and the top magnetic film, the nanocavity-shaped architecture already exhibits a unique property of efficiently capturing and storing incident radiation. Because the permeability *μ*_1_ of the GdFe material is far greater than 1 and the permeability is *μ*_2_ for non-magnetic materials, a relation of $\frac{\varepsilon_{1}\mu_{2}-\varepsilon_{2}\mu_{1}}{\varepsilon_{1}-\varepsilon_{2}}>1$ can be expected. It should be noted that a wave-vector *k* ($k_{0}\sqrt{\frac{\varepsilon_{1}\varepsilon_{2}}{\varepsilon_{1}+\varepsilon_{2}}\frac{\varepsilon_{1}\mu_{2}-\varepsilon_{2}\mu_{1}}{\varepsilon_{1}-\varepsilon_{2}}}$) under the condition of using special magnetic materials will be far more than ($k_{0}\sqrt{\frac{\varepsilon_{1}\varepsilon_{2}}{\varepsilon_{1}+\varepsilon_{2}}}$) when only using non-magnetic materials, thus resulting in a shorter wavelength of the surface plasmon generated.

It is well known that both the electric field and magnetic field components are tightly interrelated or coupled into an entire electromagnetic wavefield transporting or even memory-staying in a specified spatial region. Although the magnetic field energy is lower than the electric field component, the lightwave energy state can still be greatly enhanced by mainly increasing or resonantly accumulating magnetic fields based on an intrinsic mechanism dominated by traditional Maxwell electromagnetic relation. In other words, the light fields can also be precisely modulated or remarkably enhanced only through efficiently manipulating the magnetic field component, which may guide a better process for efficiently manipulating lightwaves and simultaneously reduce the system burden. As shown in Figure 2a, a thin Ag film contacted directly with the GdFe material pre-deposited over an n-typed Si wafer also acts as a mirror to sufficiently reflect IR lightwaves incident upon its surface into the nanocavity again, so as to further enhance the spatial interference in the nanocavity and thus remarkably decrease the radiation transmission of the metasurface developed. Currently, many conventional metallic materials such as Au or Ag or Cu [24] are suitable for fabricating the bottom reflector. So, Ag with a weak diamagnetism is selected for fabricating the bottom film according to our mature technology.

## 3. Results

### 3.1. Layered Magnetic Response Architecture

**Figure 3:**
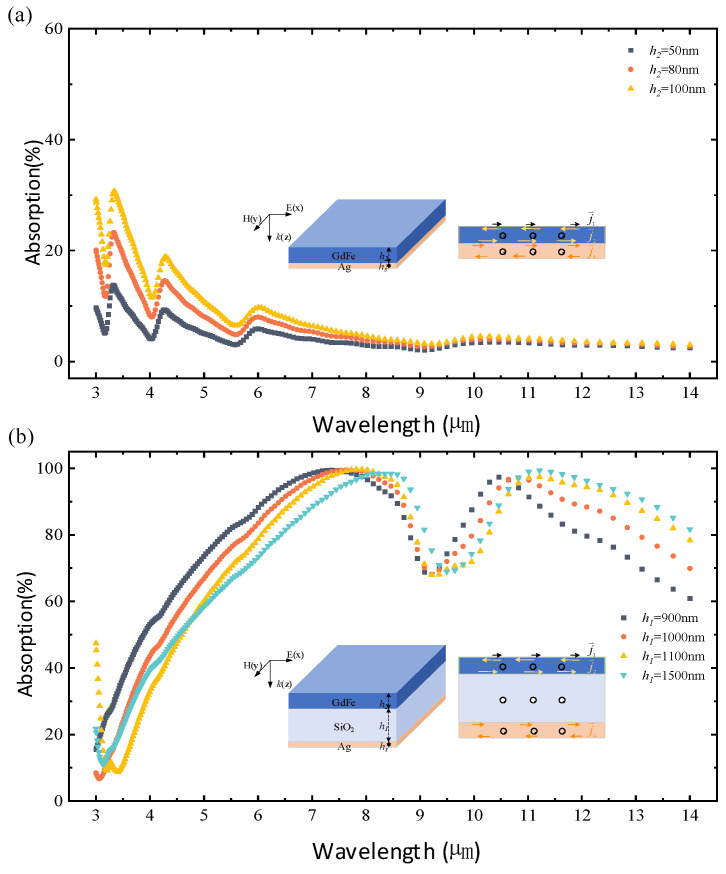
Simulations of the IR absorption of the GdFe-Ag film system and GdFe-SiO_2_-Ag nanocavity-shaped metasurface. (**a**,**b**) Spectral absorption characteristics of the GdFe films with different thicknesses designed and the GdFe-SiO_2_-Ag configuration based on different SiO_2_ thicknesses, respectively.

Typical simulations of the IR absorption of both the GdFe/Ag composite film and the GdFe-SiO_2_-Ag nanocavity-shaped metasurface are shown in Figure 3. The simulated IR absorption data are directly acquired by removing the IR reflectance and transmittance from the incident IR power. An obvious comparison according to the average IR absorption level, such as a very low value of ~13% to the GdFe/Ag composite film with a relatively high value of ~65% to the GdFe-SiO_2_-Ag nanocavity-shaped metasurface, can be observed clearly. As shown in Figure 3a, the simulated IR absorption spectrum of the GdFe/Ag composite film configurated by an optimal Ag thickness of 10 nm and the different GdFe thickness, including 50 nm, 80 nm, and 100 nm, present a similar variance trend, which begins from an initial oscillating descent in a wavelength range of 3–9 μm to a relatively stable presentation of ~4% in a wavelength range of 9–14 μm. Three absorption curves start from different initial values in a sequence of {50 nm—black} < {80 nm—red} < {100 nm—yellow} and also demonstrate an almost identical interval of ~10% at a 3 μm wavelength. It can be obtained that a suitable thickness for GdFe film should be 100 nm to remarkably reduce the surface reflectance.

Next, a SiO_2_ dielectric layer with a needed thickness is further added into the structure indicated by Figure 3a, and the absorption curves obtained by changing the SiO_2_ thickness from the initial 900 nm to 1000 nm and then 1100 nm and the final 1500 nm are shown in Figure 3b. IR absorption is significantly increased after adding a SiO_2_ dielectric layer between GdFe and Ag films. Specifically, the average IR absorptivity reaches a relatively high value of 65.45% corresponding to having 900 nm thick SiO_2_, while the average IR absorptivity corresponding to a thickness sequence of {1000 nm, 1100 nm, 1500 nm} of SiO_2_ film is in a sequence of {59.49%, 55.79%, 56.68%}. In addition, the absorption spectra of the monolayer electric–magnetic composite films are decreased as a whole with a continuous increase in SiO_2_ thickness, and the frequency points corresponding to the wave peaks of the absorption spectra will present an obvious red-shifting, but the amplitude will decrease rapidly when the film thickness is increased from 900 nm to 1000 nm. It is worth pointing out that there are two absorption peaks in the near-infrared region corresponding to a SiO_2_ film thickness of 900 nm at two wavelengths of ~10.46 μm and ~7.36 μm, respectively, and the absorption peaks are as high as 97.49% and further close to 99.49%.

**Figure 4:**
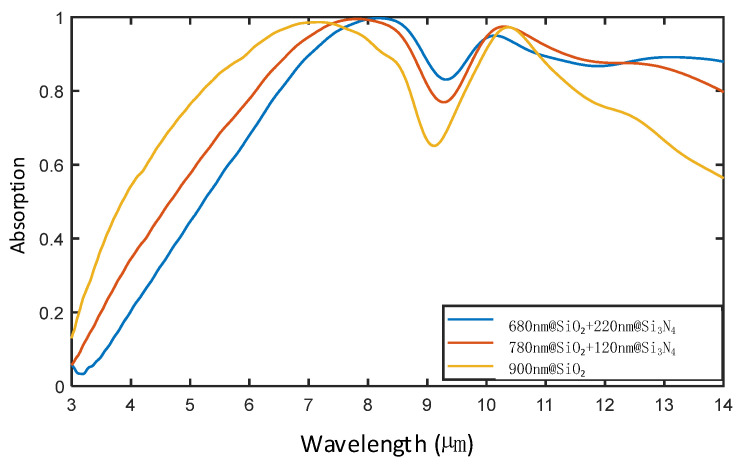
The IR absorptivity of the metasurfaces after replacing a portion of SiO_2_ with Si_3_N_4_.

As the IR absorptivity of the metasurfaces is decreased dramatically at 9 µm after adding a SiO_2_ layer, a part of the SiO_2_ material is replaced by Si_3_N_4_ material, and the calculation results for the absorptivity of the metasurface according to simulations are presented in Figure 4. Because the IR absorptivity of the metasurface is increased again at a wavelength of 9 µm with gradually increasing the thickness of Si_3_N_4_, a remarkable decrease in the IR absorptivity of the metasurface at 9 µm should be caused by the optical properties of the SiO_2_ material utilized.

**Figure 5:**
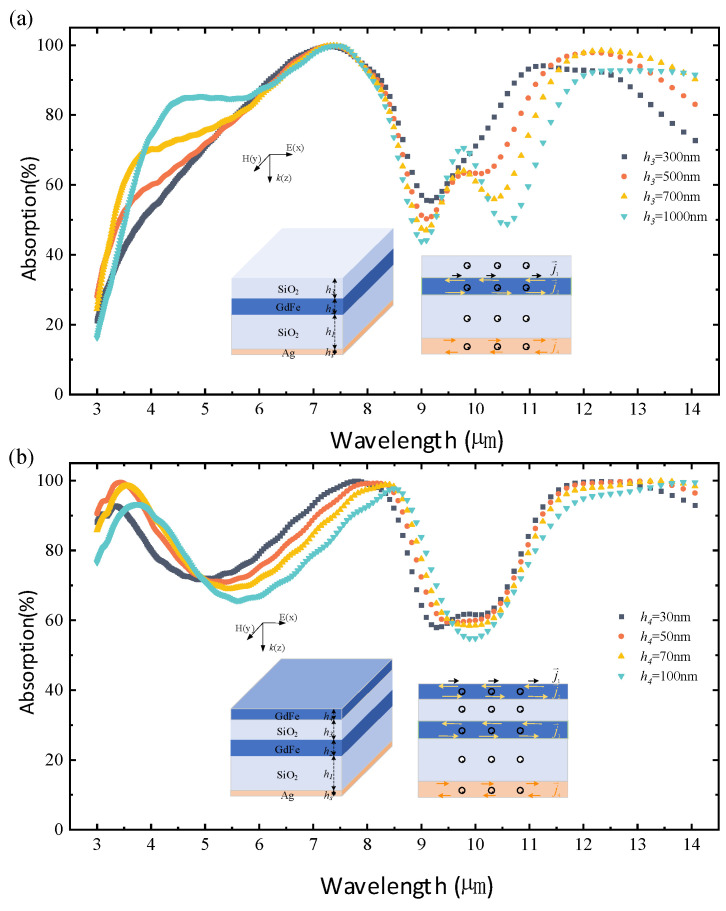
Simulations of the IR absorption of the nanocavity-shaped metasurfaces designed. (**a**,**b**) Spectral absorption characteristics of the GdFe-SiO_2_-Ag nanocavity-shaped metasurface with a top SiO_2_ layer having different thickness and a cascaded nanocavity-shaped metasurface with a top GdFe film having a different thickness, respectively.

In order to further significantly improve the IR absorptivity of the metasurface, the number of functional film layers in the layered configuration is gradually increased from a single-layer magnetic composite structure based on GdFe material on a Si wafer. As shown in Figure 5a, the addition of two SiO_2_ layers already significantly improves the IR absorptivity in the 3–7 μm band. The average IR absorptivity of the top SiO_2_ layer is ~64.61% when the thickness indicated by *h*_3_ is 300 nm, 71.24% when *h*_3_ is 500 nm, 69.32% when *h*_3_ is 700 nm, and 69.16% when *h*_3_ is 700 nm. And with the increase in the thickness, the IR absorption spectra of the magnetic metasurfaces exhibit an overall trend of initially increasing and then decreasing. When the film thickness is increased from 300 nm to 500 nm, the increase is more significant, and most of the peaks of the absorption spectra are also gradually red-shifted with the increase in the film thickness. When *h*_3_ = 500 nm, three absorption peaks of the red curve correspond to wavelengths of *λ*_1_ = 7.4 μm and *λ*_2_ = 9.61 μm and *λ*_3_ = 12.64 μm, respectively, which correspond to the absorptions as high as ~99.77% and ~65.76% and ~97.73% that can be observed. Finally, a dual-nanocavity architecture is constructed by further depositing a GdFe film with different thicknesses of 30 nm or 50 nm as well as 70 nm or 100 nm over the top of the SiO_2_ layer so as to significantly reduce the spectral IR absorption of the metasurfaces, as demonstrated in Figure 5b.

**Figure 6:**
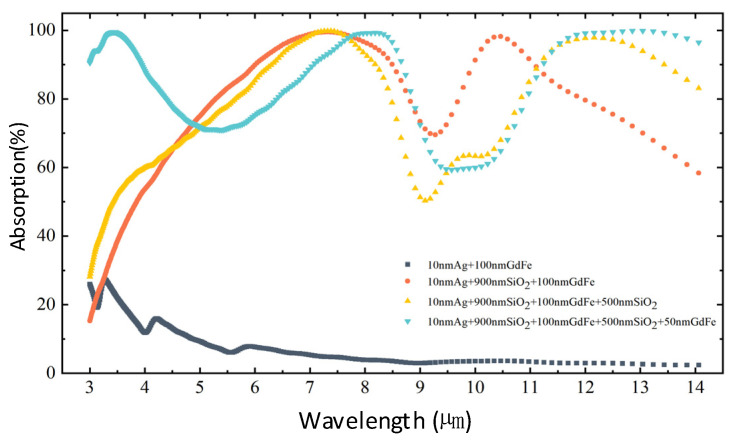
Comparison among the simulated IR absorption curves of the basic structures including a GdFe/Ag film, a GdFe-SiO_2_-Ag nanocavity, a SiO_2_/GdFe-SiO_2_-Ag nanocavity, and a cascaded GdFe-SiO_2_-GdFe/GdFe-SiO_2_-Ag nanocavity-shaped metasurface.

A comparison of the simulated IR absorption characteristics of the basic structures, including a GdFe/Ag film, a GdFe-SiO_2_-Ag nanocavity, a SiO_2_/GdFe-SiO_2_-Ag nanocavity, and a cascaded GdFe-SiO_2_-GdFe/GdFe-SiO_2_-Ag nanocavity-shaped metasurface, is given in Figure 6. As shown, in the wavelength range of 3–14 μm, the overall IR absorption of the SiO_2_/GdFe-SiO_2_-GdFe composite structure represented by a green curve is increased from ~13% to about 85% compared with that of the GdFe/Ag film represented by a black curve. This indicates that an optical nanocavity-shaped structure remarkably enhances the IR absorption efficiency through spatial magnetic field coherence. The nanocavity shaped according to the top and the bottom magnetic film configuration will firstly stimulate a type of surface “free electron” displacement current in a common surface plasmon mode over the incident surface of each magnetic film and further magnetize the GdFe material intensively, which also means that a surface equivalent eddy current over two endfaces of each magnetic film are generated effectively. A spatial magnetic field resonance mainly restricted in the nanocavity can be generated by coupling the binding eddy currents over the surface of each magnetic film contacting directly with the SiO_2_ material. So, a confined resonant enhancement in the spatial electromagnetic wavefields mainly according to the constructive interference based on a layered configuration allows for strong IR absorption in a wide wavelength range of 3–14 μm.

**Figure 7:**
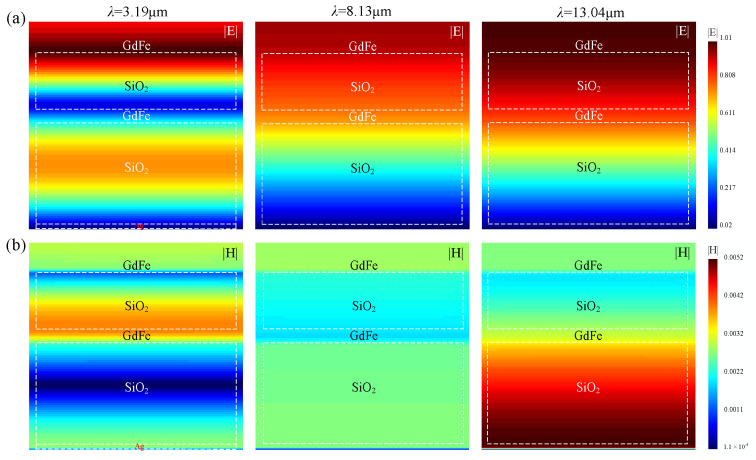
Typical simulations of the resonant electromagnetic wavefield distribution in the dual nanocavity-shaped metasurface at several featured wavelengths with an almost 100% absorption. (**a**) Electric field distribution. (**b**) Magnetic field distribution.

According to the spectral variance trend of the dual nanocavity-shaped metasurface based on a composite architecture of {GdFe/SiO_2_/GdFe} + {GdFe/SiO_2_/Ag}, an ideal spectral IR absorption with almost 100% can be observed at three wavebands, which can be indicated by three featured wavelength points of 3.19 μm and 8.13 μm and 13.04 μm selected roughly. So, the electric field and magnetic field components of incident beams are further simulated, as shown in Figure 7. The separated electric field and magnetic field components should exist in different regions and seemingly present a half-wavelength or π phase retard. In a relatively long wavelength region roughly exceeding ~11.5 μm, an instantaneous electric field mainly distributes in the top nanocavity and roughly presents a variance trend from the maximum value of 1 to the minimum value of 0.02 selected. And the magnetic fields are mainly in the bottom nanocavity and also exhibit an opposite variance trend from the initial maximum value of 1.1 × 10^−4^ at the bottom to a small value of 0.002 selected roughly. In the intermediate waveband, a similar wavefield distribution can also be observed.

The analysis of the color block variance, as shown in Figure 7, reveals that the spatial magnetic field distribution in the metasurfaces is consistent with the distribution characteristics of the common plasmon excitation, i.e., the magnetic field strength is the largest in the metal film, and the magnetic field away from the metal film will gradually decay in an exponential form. And the electric field strength in the upper SiO_2_ structure will be increased gradually with increasing the wavelength, and at the wavelength of ~13.04 μm, the electric field inside the upper SiO_2_ structure is thus extremely strong so as to indicate that the nanocavity formed between the upper and lower magnetic films already excites a surface plasmon over the surface of the magnetic film and thus generates an induced current oscillation on the upper and lower magnetic film surface of the nanocavity, respectively. Due to the displacement currents generated in the nanocavity and at the junction corresponding to the magnetic film, a strong induced magnetic moment is generated, which will confine the incident light field within the layered composite structure, leading to a strong IR absorption at ~13.04 μm wavelength. Since the magnetic and electric fields are in the form of a standing wave inside the nanocavity, the absorptivity of the metasurface can reach a peak when the wavelength of the incident light is five times the length of the nanocavity. For the simulated dual-nanocavity metasurface with a height of 1560 nm, for example, the absorptivity peaks will present at ~7.8 μm, whereas the height of the top nanocavity is 650 nm, so the absorptivity peak will present at about 3.19 μm. So, the absorption of the metasurface will present several obvious peaks near the wavelengths of 13.04 μm, 7.8 μm, and 3.19 μm, which is consistent with those shown in Figure 6.

So, the spatial distribution morphology above can be viewed as a basic intensity evolution mode (IEM) shaped by merging the electric field and magnetic field subpatterns. But in the short waveband, the spatial electromagnetic wavefields existing in the metasurface obviously present a layered character, which can be viewed as a cascaded IEMs with different amplitudes according to an intensity sequence of {E-Top Nanocavity} > {E-Bottom Nanocavity} and {H-Top Nanocavity} > {H-Bottom Nanocavity}. Generally, the total energy by integrating the electric field and magnetic field components distributed in the composite architecture above are almost the same.

**Figure 8:**
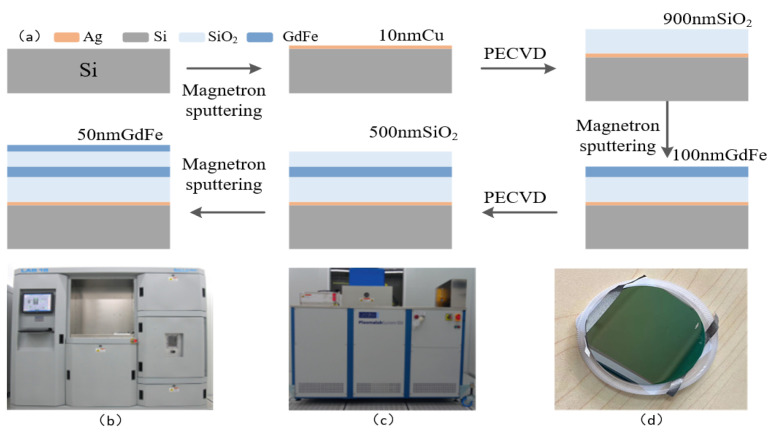
A schematic diagram of a Si-based nanocavity formed by a basic GdFe/SiO_2_/Ag film system. (**a**) Typical manufacturing process. (**b**) Sputter-Lesker-Lab18 magnetron sputtering equipment. (**c**) ICPPECVD-SENTECH-SI500 plasma-enhanced chemical vapor deposition equipment. (**d**) The typical surface appearance of the metasurface sample fabricated.

A schematic diagram of a basic GdFe/SiO_2_/Ag film system leading to a Si-based nanocavity-shaped metasurface is shown in Figure 8. The main technological process for preparing the key GdFe film involves two steps: magnetron sputtering (PVD) [25] and plasma-enhanced chemical vapor deposition [26] (PECVD) [27,28], as shown in Figure 8a. Generally, the adhesion between SiO_2_ and Ag is relatively weak. In order to enhance their adhesion, a 5 nm thick Gr film is firstly sputtered as an intermediate adhesion layer for increasing the adhesion before performing the magnetron sputtering of the Ag film with a 10 nm thickness. And then, a SiO_2_ dielectric layer is grown by PECVD and subsequently a GdFe film deposited by similar magnetron sputtering using a GdFe alloy target of 99.9% purity (Gd:Fe = 26:74, *Φ*76.2 × 3 mm). The above operation will obviously enhance the performance of the composite films, followed by the application of PECVD to deposit the corresponding thickness of SiO_2_, and finally PVD is applied again to complete the preparation of the uppermost GdFe layer. The magnetron sputtering coating equipment is Sputter-Lesker-Lab18 (USTC Center for Micro- and Nanoscale Research and Fabrication), as shown in Figure 8b. The plasma-enhanced chemical vapor deposition coating equipment is ICPPECVD-SENTECH-SI500 (USTC Center for Micro- and Nanoscale Research and Fabrication), as indicated in Figure 8c. The created samples are presented in Figure 8d.

**Figure 9:**
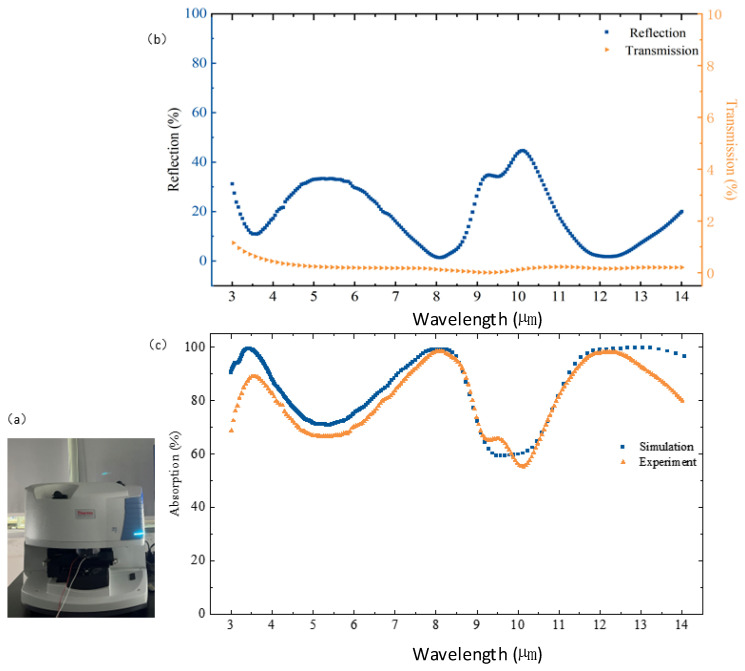
(**a**) Nicolet iN10 FTIR spectrometer (Huazhong University of Science and Technology Analytical and Testing Center). (**b**) The measured reflectance and transmittance of the Si-based GdFe metasurface. (**c**) Both the simulated and measured IR absorption characteristics of the Si-based nanocavity metasurface.

Both the simulated and measured IR absorption characteristics of the Si-based nanocavity-shaped metasurface are obtained by directly removing the reflection (reflectance, *R*) and transmission (transmittance, *T*) from incident radiations. The IR absorption characteristics of the samples are analyzed by carefully evaluating the variance in transmitted and reflected radiations according to the incident light using a Fourier transform infrared spectrometer of Nicolet iN10 (Huazhong University of Science and Technology Analytical and Testing Center), as shown in Figure 9a. The test results are shown in Figure 9b, where the blue curve indicates reflectance and the orange colour indicates transmittance. So, the absorptance is calculated by 1-*R*-*T*, as presented in Figure 9c. The graph already exhibits three distinct absorption peaks at the wavelength points of {~3.52 μm, ~8.09 μm, ~12.19 μm} corresponding to the absorption of {~89.1%, ~98.63%, ~98.23%}.

**Figure 10:**
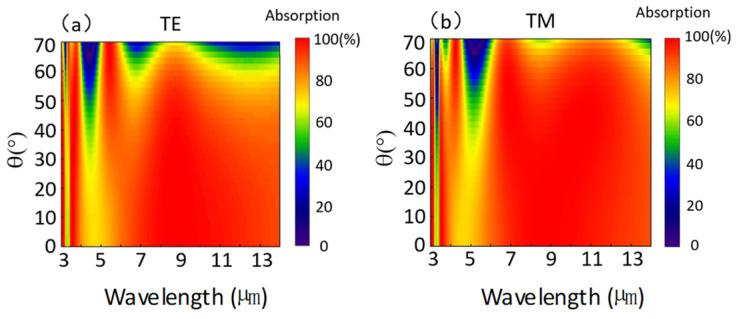
Simulations of spectral IR absorption according to incident angle θ under two polarized TM and TE modes. (**a**) The spectral IR absorption of the TE mode (**a**) and the TM mode (**b**) when varying the incident angle θ in a range of 0–70°.

The IR absorption of the GdFe film as a layered composite utilized by us generally exhibits polarization insensitivity due to a structural symmetry in the x- or y-direction mentioned above. The IR absorption behaviors under two polarized TM and TE modes to incident angle θ varied in a range of 0–70° are further simulated, as illustrated in Figure 10. So, the spectral absorption graphs can be divided by a dotted line at ~30° selected roughly according to the incident angle of the TE and TM components. When θ is less than 30°, the spectral absorption demonstrates a fairly uniform distribution with a normalized absorption of 1 indicated by the color scale attached in the wavelength range of 3.19–14 μm. After exceeding the 30° line, the spectral absorption will present an oscillation trend based on a featured incident angle indicating the IR absorption shutoff as gradually increasing the wavelength, which is demonstrated in Figure 10a for the TE mode and Figure 9b for the TM mode. Actually, the spectral absorption oscillation according to the incident angle already happens from the wavelength point of ~3.19 μm, demonstrated by a relatively low average absorption of 0.61. Both the TE and TM components of the incident IR beams still seemingly present a half-wavelength or π phase retard.

As shown, a type of bidirectional metasurface with a broadband and further narrowband radiation absorption corresponding to the top and the bottom surfaces based on alternating overlapping dielectric layers and metal films [29] has been proposed by Wang et al. When the lightbeams are incident upon the metal layer in the +z direction, the metasurface acts as a narrowband absorber and achieves 99.9% absorption at 771 nm and is then incident upon the dielectric layer in the −z direction as a broadband absorber, thus achieving a stable absorption of more than 90% in a relatively wide wavelength range from 500 nm to 1450 nm. Compared with their work, the double-layer coupled magnetic nanocavity-shaped metasurfaces proposed in this article already present excellent absorption performance in a wider wavelength range of 3~14 μm.

### 3.2. Patterned GdFe-Based Nanocavity-Shaped Metasurface

Considering the case that the spatial magnetic fields existing in the nanocavities mentioned above are generated mainly by the equivalent eddy currents surrounding each SiO_2_ layer between two adjacent GdFe films, as shown in Figure 2b, an arrayed GdFe micro-diamond cap shaped by patterned segmenting an entire GdFe film leading to a new IMAM architecture is further proposed. The spatial magnetic fields can be continuously enhanced by time-varying electric fields originated from the net charge couple inducted and redistributed locally over the GdFe and Ag films, respectively. Generally, the magnetic induction intensity **B** can be tremendously increased around a single pointed top of a basic or element magnetic micro-nano-structure design. And a very high tip density of both the positive and negative net charges compressed towards two opposite tips can be expected. So, the above factors for remarkably enhancing the spatial electromagnetic wavefields will point to an ideal prospect of further regulating the incident IR radiation through the contribution of the patterned GdFe-based nanocavity-shaped architecture by sufficiently generating and then strengthening spatial time-varying electric fields.

**Figure 11:**
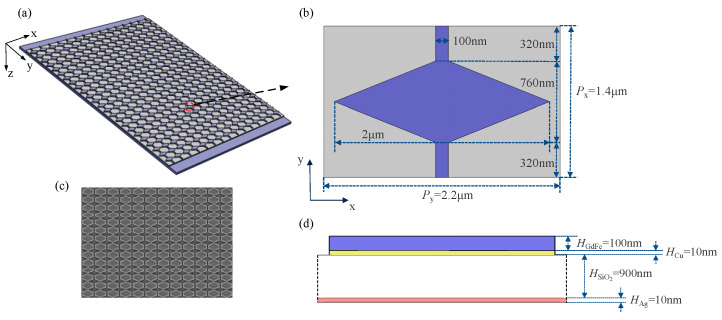
A new type of GdFe-based nanocavity-shaped metasurface. (**a**) The typical layout of the GdFe/Cu micro-diamond cap array over a SiO_2_ dielectric layer. (**b**) An element micro-diamond cap with several key structural parameters. (**c**) A SEM photograph of the top patterned appearance of the metasurface fabricated. (**d**) A cross-sectional view of a single GdFe nanocavity.

So, a thin Cu film of 10 nm thickness is attached over the backside of the top GdFe film with 100 nm thickness, which is fabricated by a conventional technological process. And a GdFe/Cu micro-diamond array is shaped through maintaining an effective wire connection between adjacent GdFe/Cu micro-diamonds along the x-direction and further connecting each cluster of the GdFe/Cu micro-diamonds over two terminals along the y-direction. A new type of GdFe-based nanocavity-shaped metasurface based on an arrayed GdFe/Cu micro-diamond cap is shown in Figure 11. The layout of the GdFe/Cu micro-diamond cap array over a SiO_2_ dielectric layer is shown in Figure 11a, and a basic or element micro-diamond cap is also shown with key structural parameters including the period P_x_ = 2.2 μm and P_y_ = 1.4 μm and both a long and short diagonal length of 2 μm and 760 nm. The adjacent micro-diamond caps are connected by a rectangular strip with a width of 100 nm and a length of 640 nm. A cross-sectional view of a single GdFe nanocavity from a patterned GdFe-based nanocavity-shaped metasurface shown by a SEM photograph in Figure 11c is given in Figure 11d. A single GdFe/Cu cap is thus coupled with a bottom Ag film of 10 nm thickness, which also acts as a reflector, so as to form a semi-opened nanocavity filled fully by a SiO_2_ dielectric layer with a thickness of 900 nm.

**Figure 12:**
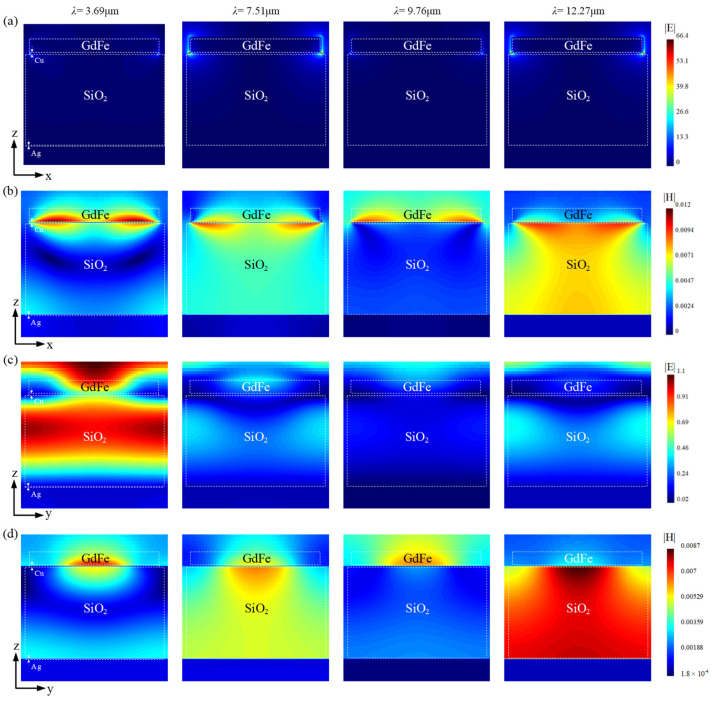
Typical simulations of the spatial electromagnetic wavefield distribution corresponding to a patterned GdFe-based nanocavity-shaped metasurface at several featured wavelength points of ~3.69 μm, ~7.51 μm, ~9.76 μm, and ~12.27 μm, respectively. (**a**,**c**) Spatial electric field distribution along the x- and y-direction. (**b**,**d**) Spatial magnetic field distribution along the x- and y-direction.

Typical simulations of the spatial electromagnetic wavefield distribution corresponding to a patterned GdFe-based nanocavity-shaped metasurface at several featured wavelength points of ~3.69 μm, ~7.51 μm, ~9.76 μm, and ~12.27 μm are shown in Figure 12. The spatial electric field and magnetic field components are displayed along the x- and y-direction, respectively.

As shown in Figure 12a, two bright points with different intensities including a maximum value of ~66 at the wavelength points of ~7.51 μm and ~12.27 μm, which are located at two opposite tips of each GdFe/Cu micro-diamond along the x-direction, also reveal the net charges as the sources of the spatial electric field mainly distributed over the charged Cu film. The relatively weak linear electric field over each apex of a GdFe/Cu composite mask should be generated by a couple of inducted charges located at the tip of the Cu film and the upper apex of the GdFe film. As shown by Figure 12b, the spatial magnetic field should be composed of two identical parts with a similar appearance corresponding to a top GdFe/Cu micro-diamond and further two similar patterned parts in SiO_2_ medium cavities on the both sides of a micro-diamond along the x-direction. So, the appearance of the top spatial magnetic fields can be attributed to a couple of conductive currents towards or away from the central region of a single micro-diamond, leading to a patterned net charge distribution above, and thus present a spectral intensity sequence of {~12.27 μm} > {~7.51 μm} > {~3.69 μm} > {~9.767 μm}. In the y-direction, the spatial electric fields are already divided into two parts by the Cu film. The electric field distributed over the Cu film should be generated by the central net charges of a single micro-diamond, and those existing between the SiO_2_ medium cavities mainly originated from the inducted net charges located at the apexes of the GdFe/Cu micro-diamond. According to the measurements and further evaluation, a similar spectral intensity sequence of {~9.767 μm} < {~7.51 μm} < {~12.27 μm} < {~3.69 μm} can be obtained. The spatial magnetic fields that originated from the inductive currents excited over the GdFe/Cu film and also the Ag film are given in a similar spectral intensity sequence of {~9.767 μm} < {~3.69 μm} < {~7.51 μm} < {~12.27 μm}. By the conducted simulations above, a total memory-resident electromagnetic wavefield distribution attributed to a patterned GdFe-based nanocavity-shaped metasurface can be ranked in a spectral intensity sequence of {~9.767 μm} < {~7.51 μm} < {~3.69 μm} < {~12.27 μm}.

**Figure 13:**
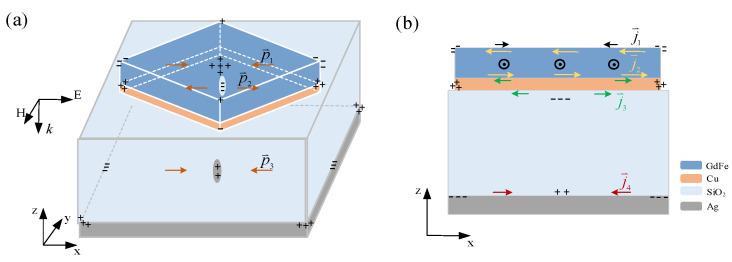
Typical distributing characteristics of both the surface net charge and conductive current as the sources of the spatial electromagnetic wavefields constrained by a patterned GdFe-based nanocavity-shaped metasurface. (**a**) Charged GdFe/Cu and Ag films along the x- and y-direction, respectively. (**b**) Surface inducted currents including bounding eddy currents for exciting spatial magnetic fields only along the x-direction in the GdFe/Cu nanocavity.

**Figure 14:**
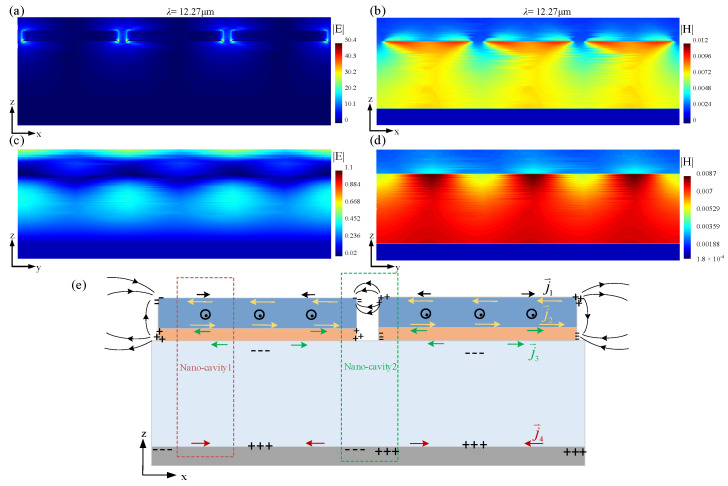
A schematic diagram of the basic spatial electric field and magnetic field appearances at a typical wavelength of 12.27 μm along the x- and y-direction, leading to an electromagnetic plasmon resonance in the z-plane. The basic fashion formed by integrating three basic electric fields (**a**,**c**) and magnetic fields (**b**,**d**), and a transient surface net charge and current morphology of a partial GdFe nanocavity (**e**).

The typical distributing characteristics of both the surface net charge and conductive current, as the sources of the spatial electromagnetic wavefields corresponding to the patterned GdFe-based nanocavity-shaped metasurface, are shown in Figure 13. A transient charged fashion of a single GdFe/Cu micro-diamond cap and a bottom Ag film is illustrated in Figure 13a. As shown, a couple of the central orientated electric dipoles **P**_1_ stimulated by the surface plasmons excited through a beam of IR radiation incident upon the top surface of the GdFe film will induct other electric dipoles **P**_2_ out from the same negative net charges located at the central region of the Cu mask and continuously a couple of the relatively weak electric dipoles **P**_3_ having the same orientation with that over the top surface along the x-direction. And there is a relatively strong and weak alternate arrangement of the positive net charges according to the central charge distribution over a single micro-diamond and further two inducted linear arrangements over both the Cu and Ag films along the y-direction, respectively. A transient surface conductive current morphology of a single GdFe nanocavity is illustrated in Figure 13b. As shown, a couple of the conductive currents **J**_1_ with two opposite directions colored by black are also stimulated by the surface plasmons excited from the incident IR beams, then the yellow surface eddy current **J**_2_ inducted by the incident magnetic field component, the surface inducted current **J**_3_ towards two tips over two endfaces of the Cu film colored by green, and then the red conductive current **J**_4_ having an opposite direction of that colored by green over the upper surface of the bottom Ag film only along the x-direction. So, the spatial electromagnetic wavefields can be resonantly accumulated and greatly enhanced in the nanocavity according to constructive interference leading to the spatial magnetic plasmon, which may imply a new IR radiation response and absorption manner being different with the conventional irreversible optothermal sensing and absorbing techniques. A typical fragment corresponding to a spatial plasmon mode with a periodic fashion from the resonant spatial electromagnetic wavefields integrated by the electric field and magnetic field components is shown in Figure 14. Both the basic spatial electric field and magnetic field appearances at a typical wavelength of 12.27 μm along the x- and y-direction, which are formed by assembling three basic fashions from Figure 12, are shown in Figure 14a,b, respectively. A transient surface net charge and current morphology of a partial GdFe-based nanocavity-shaped metasurface involving a basic sealed nanocavity and also a basic semi-opened nanocavity directly exposing the SiO_2_ material to the incident radiation, which can be classified as two types of nanocavity architectures, is further illustrated in Figure 14e.

**Figure 15:**
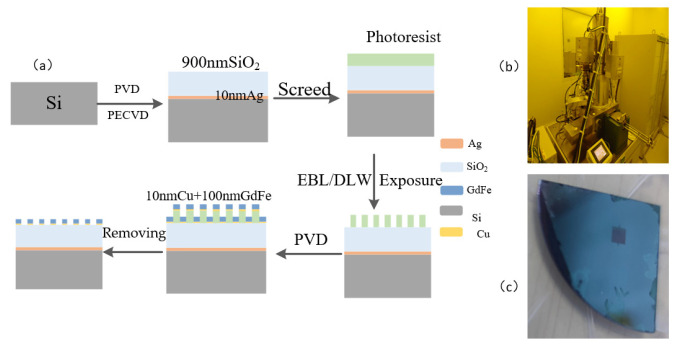
(**a**) Process preparation flow for silicon-based GdFe nanorhombic array magnetic supersurfaces. (**b**) EBL-JEOL-6300 Electron Beam Lithography Equipment.(USTC Center for Micro- and Nanoscale Research and Fabrication.) (**c**) Nanorhombic array magnetic hypersurface.

The technological flow for the preparation of Si-based GdFe nanorhombic array magnetic metasurfaces mainly includes the following: magnetron sputtering coating (PVD), plasma-enhanced chemical vapor deposition (PECVD), direct laser writing (DLW)/electron-beam lithography (EBL), magnetron sputtering coating (PVD), and removing photoresist film or masks, as shown in Figure 15a. The new IMAM architecture is fabricated by adding a crucial step of the EBL of EBL-JEOL-6300 demonstrated in Figure 15b. After completing basic operations such as electron beam exposure and development and fixation for defining the structural pattern, the subsequent steps involving the sputtering deposition of GdFe magnetic film followed by common ultrasonic treatment are performed. A methodical process will ensure meticulous separation between the magnetic film and the photoresist, so as to result in an effective creation of the GdFe micro-diamond array. The final sample is exhibited in Figure 15c.

**Figure 16:**
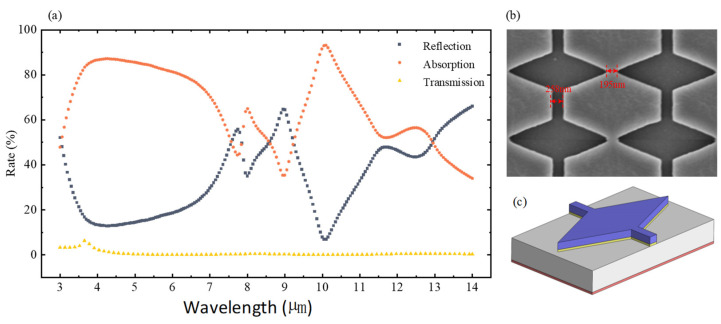
Typical characteristics of the patterned IMAM sample acquired by us. (**a**) The measured optical response characteristics of the sample. (**b**) A SEM photograph of the patterned IMAM sample fabricated. (**c**) A 3D view of a single GdFe/Cu micro-diamond cap.

The typical characteristics of the patterned IMAM sample acquired by us are shown in Figure 16. A basic GdFe/Cu micro-diamond cap and a SEM photograph of a partial sample are illustrated in Figure 16b,c, respectively. The IR absorption characteristics in the wavelength range of 3–14 μm are given in Figure 16a.

As shown, the overall average IR absorption is ~71.7%, which is much lower than that shown in Figure 9c because of the intrinsic SiO_2_ absorption, which is roughly consistent with that indicated at the wavelength of 9.76 μm in Figure 12. The overall transmittance is almost zero with an overall reflectance of ~30%. It can be noted that the patterned IMAM already achieves a relatively stronger IR absorption, because the joint action of the surface oscillated net charges distributed over the charged metallic films and the surface conductive currents including eddy currents will generate stronger spatial electromagnetic wavefields in an arrayed nanocavity-shaped architecture under the condition of completely eliminating SiO_2_ absorption.

## 4. Conclusions

A type of IMAM consisting of the key GdFe films and SiO_2_ dielectric layers is proposed for realizing ideal IR lightwave manipulation and absorption mainly based on spatial electromagnetic wavefield excitation and resonant accumulation. The simulations and measurements demonstrate that the GdFe-based nanocavity-shaped metasurfaces already achieve an average IR absorption of ~81% in a wide wavelength range of 3–14 μm, experimentally. It can be expected that the joint action of the surface oscillated net charges distributed over the charged metallic films and the surface conductive current including equivalent eddy currents will generate strong spatial electromagnetic wavefields by constructing a patterned surface metallic micro-diamond array and the film system leading to a nanocavity-shaped array. It should be noted that the IR lightwave manipulating and memory-resident absorbing in an electromagnetic storage manner can be further improved through continuously optimizing the metallic and medium material configuration and also the patterned surface layout based on the joint action of the spatial electric field and magnetic field components stimulated in the nanocavity-shaped architecture. This work highlights several potential applications such as highly efficient thermal radiation responding and sensing, IR radiation manipulating and re-arranging for detection, and miniaturized photonic devices.

## Data Availability

Data are contained within the article.
